# EAHE-D: A dataset for modeling and performance evaluation of earth to air heat exchangers

**DOI:** 10.1016/j.dib.2024.111110

**Published:** 2024-11-05

**Authors:** Youssef Alidrissi, Marouane Wakil, Mehdi Najib, Samir Idrissi Kaitouni, Mohamed Oualid Mghazli, Mohamed Bakhouya

**Affiliations:** a International University of Rabat, College of Engineering and Architecture, LERMA Lab & TICLab, Sala Al Jadida, Morocco; b Green Energy Park (IRESEN,UM6P), km2 R206 Benguerir, Morocco

**Keywords:** Energy efficient buildings, Thermal comfort, Earth to air heat exchanger, Simulations and performance evaluation, Energy modeling, Passive design, NZEB

## Abstract

Integrating natural ventilation with Earth-to-Air Heat Exchangers (EAHE) is an innovative approach that effectively combines the benefits of both systems to enhance indoor environments while reducing energy consumption and operational costs. Many studies focus on reducing reliance on conventional heating, ventilation, and air conditioning systems by optimizing the use of EAHE and natural ventilation in buildings to improve occupant comfort. However, these studies require actual datasets for systems’ design and optimization. This work aims to provide such a dataset using a deployed EAHE with in-situ measurements. The dataset is available in its original form, allowing users to perform data preprocessing according to their specific needs. Additionally, we offer a processed version of the dataset, which can be used to further investigate the effectiveness of EAHE deployment in buildings and its integration with other passive and active systems, aiming to enhance energy efficiency while maintaining occupant comfort.

Specifications Table

**Table:** 

Subject	Energy and Buildings, Passive/active systems, Data monitoring and processing, Modeling and simulations
Specific subject area	Earth to air heat exchanger.
Type of data	Table.
Data collection	An EAHE prototype has been deployed and monitored in real-time using six temperature sensors. An embedded system collects, each 1 min, the data from sensors and stores it internally and remotely for being used in modeling and performance evaluation when integrated into the building. Sensors are placed as follows. Two of them measure Inlet and outlet air temperatures, while the others are measuring both air and ground temperatures. The dataset is composed of raw data sets in the form of CSV files. It is shared in its original version to allow those interested to perform data preprocessing according to their own needs. We also offer a processed version of this dataset. More precisely, dataset is not processed, missing and unwanted values are maintained, especially for persons willing to test interpolation approaches [1].
Data source location	● Institution: Green Energy Park (IRESEN,UM6P) ● City/Region: km2 R206 Benguerir ● Country: Morocco ● GPS coordinates: 32°14′20.9″N latitude and 7°57′14.2″W longitude
Data accessibility	Repository name: zenodo.org Data identification number: 10.5281/zenodo.12820831 Direct URL to data: https://zenodo.org/records/12820831
Related research article	none

## 1 Value of the Data

• **All-Inclusive Environmental Parameters**: Involves the measurement of indoor/outdoor and ground temperatures, detailed weather data for complex modeling purposes.
• **Calibration of prediction Models**: Acts as a reference for the calibration of EAHE models thereby enhancing modeling accuracy.
• **Control Strategies Development**: Assists in the development of advanced control strategies such as model predictive control for EAHE system design.
• **Evaluation of Seasonal Performance**: Studies the seasonal performance of EAHE during winter months.
• **Integration with Renewables**: Allows the development of combined systems in which renewable resources can be effectively used for both heating and cooling.
• **Educational Resource**: Provides primary data for research and practice in engineering and green building design.
• **Baseline for Longitudinal Studies**: Establishes the basis for conducting more detailed studies of the operation of EAHE and climate change over time.
• **HVAC systems design:** Companies involved in manufacturing and designing HVAC systems can utilize the dataset to optimize system designs, improve energy efficiency in buildings, reduce operational costs, and enhance occupant comfort.
• **Energy policy implementation:** The Moroccan Agency for Energy Efficiency (AMEE) can use the dataset to support its mission of implementing government policies aimed at reducing energy dependency by promoting energy efficiency initiatives, thereby informing strategies and programs that enhance sustainable energy practices.

## 2 Background

Buildings account for >40 % of total energy consumption and nearly about 40 % of greenhouse gas emissions. This significant portion is expected to rise in the coming years due to the growing use of household appliances and devices aimed at enhancing living standards and meeting comfort needs. It is crucial, therefore, to mitigate buildings' energy usage and cut down on their emissions while ensuring occupants comfortable conditions. Achieving this requires approaches in order to balance between reducing energy consumption, maintaining occupant comfort, while reducing greenhouse gas emissions [2].

Most studies showed that the energy consumption increase is due to the heavy usage of Heating, Ventilation and Air Conditioning (HVAC) systems [[3], [4], [5], [6]]. Passive systems have been all time investigated for being integrated into buildings, mainly during construction, such as wind towers, earth to air heat exchanger, evaporative systems. For instance, earth to air heat exchangers (EAHE) show significant promise for both preheating air in winter and precooling it in summer, ensuring thermal comfort within indoor zones [7]. Essentially, fresh air passes through buried pipes, exchanging heat with the ground or soil temperature. During summer, the air is cooled, thereby minimizing the need for cooling. Conversely, in winter, the process is reversed, leveraging the ground's consistent temperature throughout the year.

Most studies investigated and assessed the performance and effectiveness of EAHE in reducing energy consumption while maintaining occupants' thermal comfort within site-specific dynamics of buildings. However, design and dimensioning site-specific EAHE required accurate modeling and calibration, which requires a dataset of already deployed systems. So, this requirement was one of the objectives behind the deployment of a small EHAE system and the generation of the EHAE-D dataset. Building dataset in the Moroccan context and specific locations (Ben Guerir, Morocco climate zone 5) is highly required to assess related buildings’ services (e.g., heating, cooling, and air-quality).

The EAHE-D dataset was first used to assess a decision-making algorithm, which is designed to optimize the use of EHAE and ventilation in buildings, aiming to improve thermal comfort and reduce as much as possible the reliance on traditional HVAC systems [8].

## 3 Data Description

In its original version, as described in Table 1 and csv file “geoThermal_DS1.csv”, the EAHE dataset is timestamped using Unix epochs, as is common in Raspberry Pi boards. Meteorological data, from a weather station located in the same area, has sophisticated abilities to monitor many weather features, among them Global Horizontal Irradiance (GHI), Diffuse Horizontal Irradiance (DHI), and Direct Normal Irradiance (DNI). In addition, it tracks the surrounding temperature, humidity, speed and direction of the wind, amount of rainfall, and atmospheric pressure, and is also provided with high-frequency sampling, at a frequency of one minute “weatherStation_DS1.csv”, as also described in Table 2.

**Table 1:** Temperatures data files' content.

Parameter	Definition	Unit
epoch	From Dec. 2023 till March 2024	epoch
IN	Inlet sensor	Degree (°C).
OUT	Outlet sensor	Degree (°C).
AirLeft	Left underground/air sensor	Degree (°C).
AirRight	Right underground/air sensor	Degree (°C).
GroundLeft	Left underground/soil sensor	Degree (°C).
GroundRight	Right underground/soil sensor	Degree (°C).

**Table 2:** Weather data files' content.

Parameter	Definition	Unit
GHI_corr_Avg	Average Global Horizontal Irradiance	W/m^2^
DNI_corr_Avg	Average Direct Normal Irradiance	W/m^2^
DHI_corr_Avg	Average Diffuse Horizontal Irradiance	W/m^2^
Tair_Avg	Average Air Temperature	°C
RH_Avg	Average Relative Humidity	%
BP_CS100_Avg	Average Barometric Pressure	hPa
WS_Avg	Average Wind Speed	m/s
WD_Avg	Average Wind Direction	°
WD_Std	Wind Direction Standard Deviation	°
WSgust_Max	Maximum Wind Speed Gust	m/s

### 3.1 Data preprocessing

**Fig. 1:**
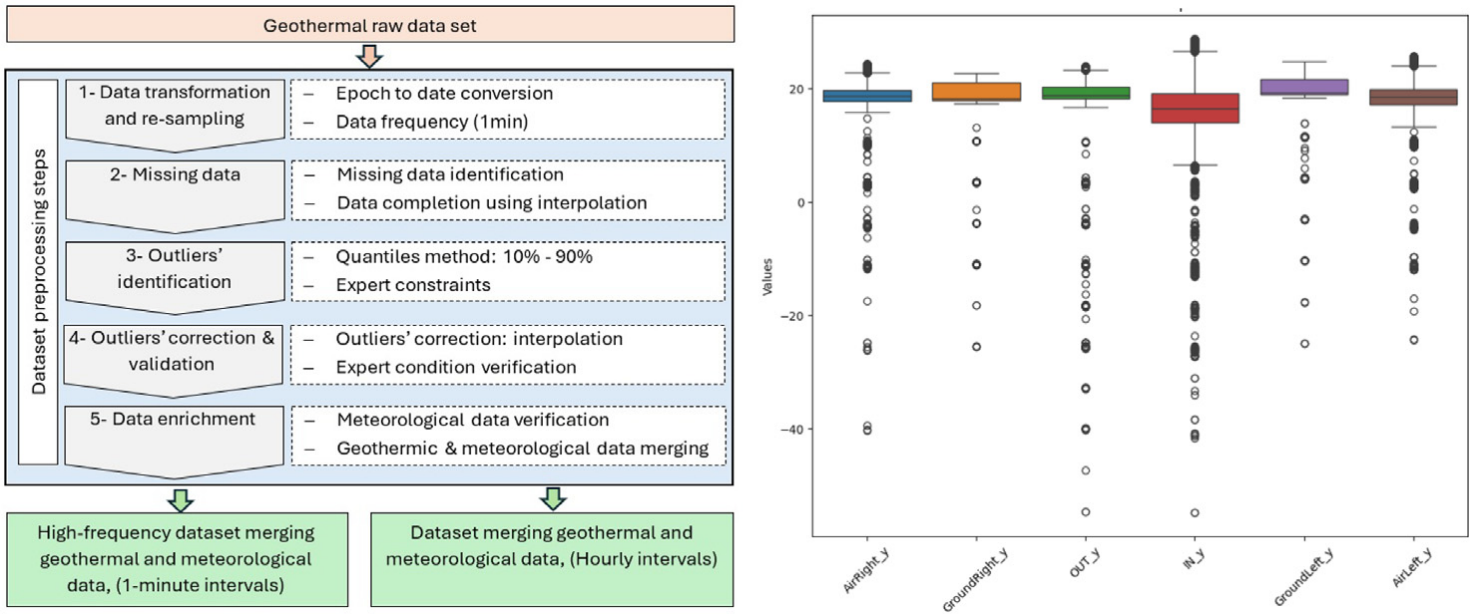
Dataset preprocessing steps together with the boxplot of the geothermal parameters for outliers’ detection (10 %–90 %).

The “geoThermal_DS1.csv” is processed following the steps depicted in Fig. 1. First, the epochs are then transformed into the format ``DD:MM:YYYY hh:mm:ss'' and sampled to obtain one record per second. Thus, multiple measurements taken within the same minute are replaced by the average of the existing measurements. Resampling at a frequency of one minute generates records without measurements. These records are identified in the second step as missing data and are replaced by interpolation, based on the parameter values recorded in the records before and after the missing interval. This step produced the files “geoThermal_DS2.csv”. The third step of our process focuses on detecting outliers for the various measurements taken. We use a method based on quantiles with a 10 % and 90 % interval (Fig. 1), based on various test and sensibility analysis, combined with a constraint defined by a geothermal expert, stating that the value of the parameter ``OUT_Y'' must be between the min (IN_Y) and max (IN_Y) recorded during the day.

**Fig. 2:**
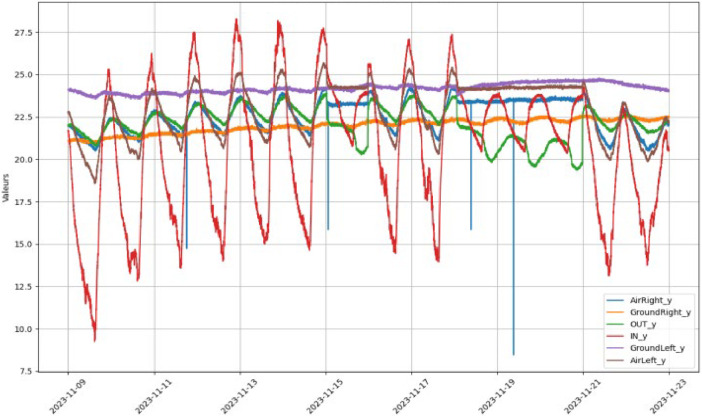
Original EAHE data set for 2 weeks.

In the subsequent step, values detected as outliers are treated as missing data and replaced by the result of interpolation. Once this process is completed, outlier detection is re-launched to ensure the validity of the values generated by interpolation. This step produced the “geoThermal_DS3.csv” file. The final step involves merging the datasets of geothermal data with meteorological data “weatherStation_DS1.csv” Holds meteorological data without any processing. The final dataset merges the geothermal and weather data, sampled at both high-frequency intervals of one minute “geoThermalWeather_minute.xls” and hourly intervals “geoThermalWeather_hourly.xls”. Fig. 2 shows the original EAHE dataset values including missing data and outliers. It is worth noting that all modifications were performed using a Python script, with the following libraries: pandas for data manipulation and computation, Seaborn and Matplotlib for data visualization. The versions of the tools used are as follows: Python: 3.10.12 (main, Sep 11, 2024, 15:47:36) [GCC 11.4.0], pandas: 2.2.2 and Seaborn: 0.13.2.

## 4 Experimental Design, Materials and Methods

This section provides more details about the deployed EHEA system. It is dedicated to a brief description of EHEA components including the deployed platform's prototype for data monitoring and processing. More detail about its design and integration into the building is described in [8].

### 4.1 *EHEA system's components*

**Fig. 3:**
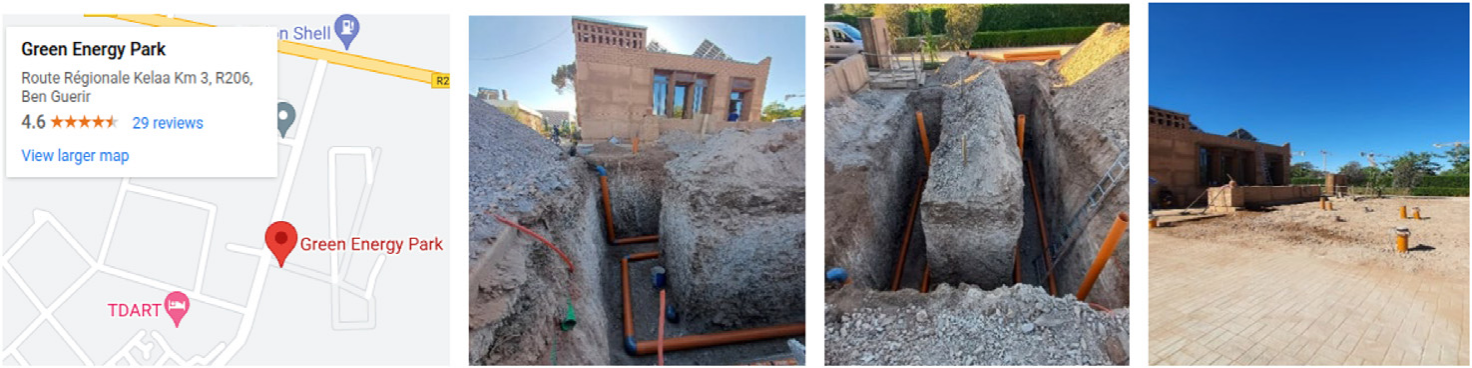
The deployed EHEA system.

The EHEA was deployed in AFRIKATATERRE house at the Green Energy Park (IRESEN, UM6P), km2 R206 Benguerir, Morocco, which is located at the coordinates 32°14′20.9″N latitude and 7°57′14.2″W longitude, as depicted in Fig. 3. The experimental setup is located on the side of the AFRIKATATERRE house, which was built to be energy and comfort efficient throughout the year. The EAHE could function as either a heater or cooler following the weather conditions. It is worth noting that the Benguerir city has semi-arid climate conditions, with 18 °C/38 °C (resp. 8 °C/22 °C) as an average daily (resp., night) temperature. It is almost dry for 267 days a year with an average humidity of 53 %. In the region, it rains almost 77 days (approximately 165 mm rainfall) with 3399 sun hours throughout the year.

The pipelines of the EAHE were placed inside two trenches of 6 m long, 1.5 m wide, and 3.5 m deep, as shown in Fig. 3. The PVC pipe, with 15 cm as diameter and 30 m total length, has been used for the canalization. The serpentine deployment layout was used, because of space limitation.

**Fig. 4:**
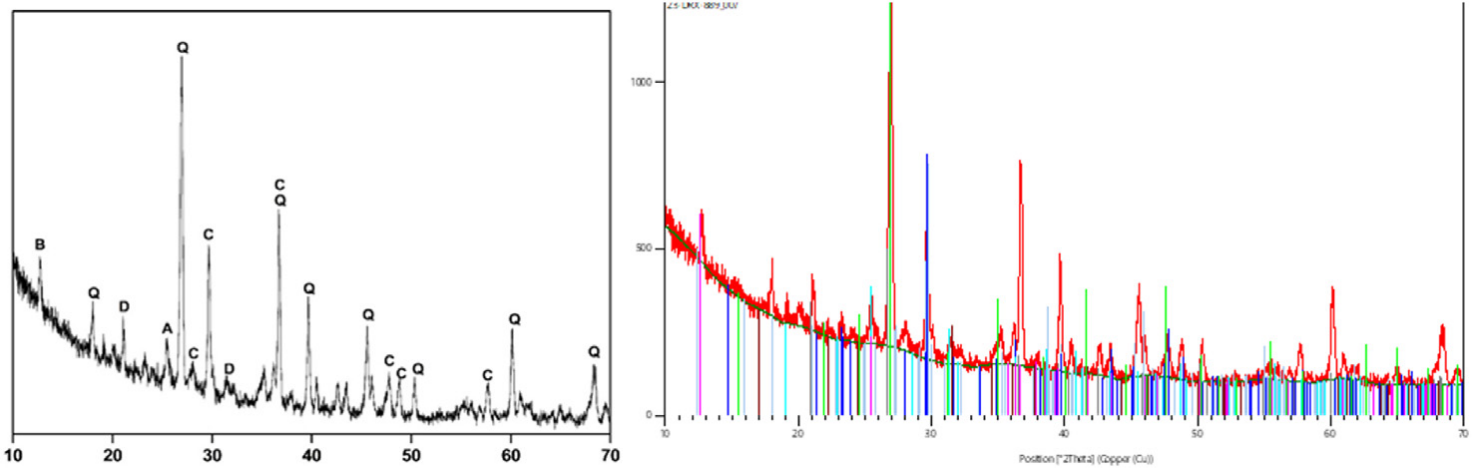
X-ray diffraction (XRD) spectrum of soil sample for mineralogical characterization.

It is worth noting that the mineralogical composition of the soil samples obtained was determined using XRD (X-ray diffraction) analysis as shown in Fig. 4. Our analysis showed a varied composition of minerals, suggesting the complex geological heritage of the earth's surface. The most prevalent mineral identified was Calcium Carbonate (CaCO3), with a match rating of 51, indicating its high prevalence and implying that the ground could have alkaline attributes. In the course of our research, we recognized significant amounts of Genthelvite, which is a complex zinc silicate, with a score of 29. Quartz, which is at a score of 19, constituted a major part, reflecting the sandy nature of the soil and implying excellent drainage qualities. Dolomite and Anhydrite were detected with values of 9 and 21, accordingly. Finally, Birnessite, having a score of 16, was recognized for its contribution to the process of manganese cycling in the soil. The XRD examination offers an exhaustive mineralogical description of the soil, which offers helpful information. Comprehending these mineral constituents helps in projecting earth performance in various environmental conditions. In addition, the thermal characteristics of the soil utilized during the process are shown in the table supplied under. Together with each other, they aid in a comprehensive evaluation of the Kusuda equation's effectiveness in predicting earth temperatures in function of underground depth, ensuring the dependability and precision of the EAHX model (Table 3).

**Table 3:** Thermophysical Properties of Soil.

Property	Value
Thermal Conductivity (W/mK)	1.93
Specific Heat (J/kgK)	1657
Density (kg/m³)	1178

### 4.2 *Platform's prototype and data monitoring*

**Fig. 5:**
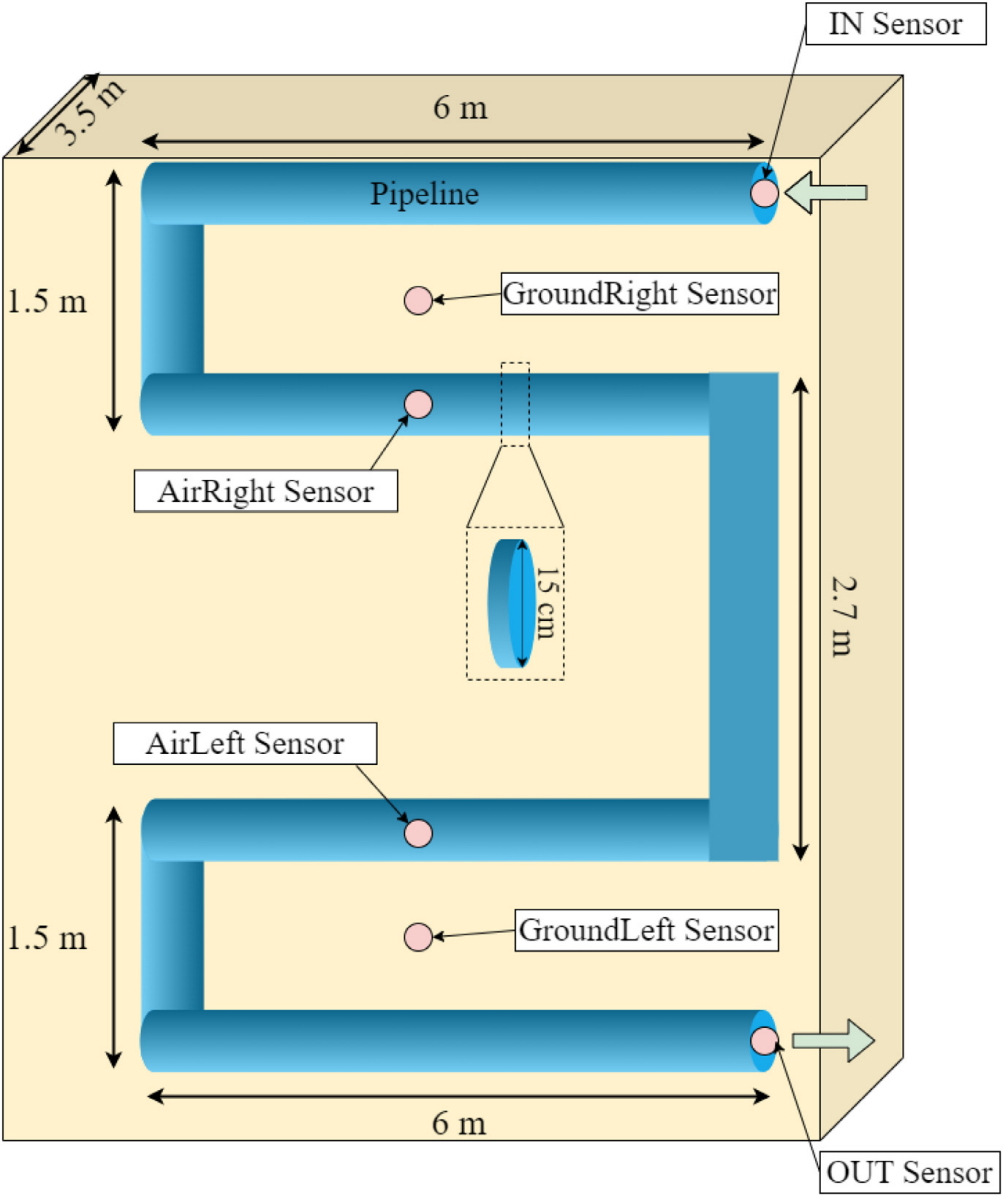
Overall system schematic illustrating the positioning of sensors.

Six temperature sensors IN, OUT, AirLeft, AirRight, GroundLeft, GroundRight were deployed. The sensors are of the same model “DS18b20 '' with stainless steel package for water resistance. The DS18b20 sensor is a digital thermometer with a built-in 12-bit ADC. It does not require additional circuits for interfacing with microcontrollers, making it a cost-effective sensor with ease of implementation. The temperature range of this sensor is adequate to the prototype and can vary between −55 °C minimum temperature to +125 °C maximum with 0.5 °C as precision. For instance, the IN sensor monitors the air temperature at the inlet, while the OUT sensor measures the temperature at the outlet of the system. AirRight/AirLeft give the underground air (inside the pipe) temperature at 3.5 m depth and at two different points alongside the pipe, while GroundLeft/GroundRight monitor the underground soil (outside the pipe). These latter have been installed separately into a copper head from inside in order to ensure good contact and heat transfer between sensors and the copper heads. The outside of the head is arrow shaped and in contact with soil. Furthermore, a 3.5 m tube was used at each underground sensing point to install the sensors, so they can be changed easily in case of damage. A ventilator is used and deployed at the system's output for air extraction being injected into the house. A schematic illustrating the exact position of each sensor in the deployed system is presented in Fig. 5.

**Fig. 6:**
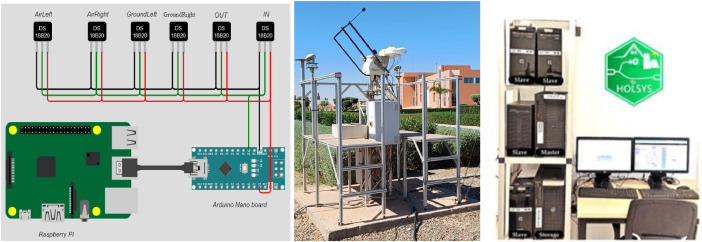
Deployed soil sensors/weather station and prototype for data acquisition.

Sensors are linked to an embedded system as shown in Fig. 6. The DS sensors are connected in external power mode using low resistance cables to prevent voltage drop and signal loss over long distances. They are connected to an Arduino Nano that provides the required power and communicates with the sensors using OneWire communication protocol. In fact, OneWire is a communication protocol designed for low-speed, low-complexity communication with microcontrollers. It allows multiple devices to communicate using a single data line, with each device having a unique 64-bit address. After the Arduino receives the temperatures data from the sensors, it sends them to a Raspberry PI 4 using Universal Serial Bus protocol. At each sampling time, the Raspberry PI sends the received data over MQTT to the HOLSYS platform's prototype, which we have developed and deployed mainly for data monitoring, processing and storage (Fig. 6). The HOLSYS platform's prototype uses recent IoT and Big data technologies including Wifi communications for remote data sensing. It is used for many scenarios for energy efficiency and smart control of building's services. The aim is to provide the Sw/Hw components that are required for real-time data collection and communication interfaces to the building's equipment.

## Limitations

None.

## Ethics Statement

The authors confirm that they have respected ethical issues (e.g., Human embryonic stem cells, Human participants, Human cells or tissues, personal data, animals, etc.) and the deployment of the EAHE system together with all tests have been conducted in a safe way for the participants. There is no data related to the participants.

## CRediT Author Statement

**Youssef Alidrissi, Marouane Wakil, Mehdi Najib, Samir Idrissi Kaitouni, Mohamed Oualid Mghazli, Mohamed Bakhouya**: Conceptualization, Methodology, Software, Data curation, Writing- Original draft preparation; Visualization, Investigation, Software, Validation, Writing- Reviewing and Editing, Supervision

## Data Availability

zenodoEAHE-D: a Dataset for Modeling and Performance Evaluation of Earth to Air Heat Exchangers (Original data)
